# Professionals' Perceptions of the Colorectal Cancer Pathway: Results of a Co‐Constructed Qualitative Study

**DOI:** 10.1111/hex.14146

**Published:** 2024-07-14

**Authors:** Matthieu Delaye, Alice Polomeni, Sylvie Faiderbe, Nadège Berlioz, Chaïma Benssekoum, Aude Guillemin, Thomas Pudlarz, Sandrine de Montgolfier

**Affiliations:** ^1^ Department of Medical Oncology, Curie Institute, Versailles Saint‐Quentin University Paris Saclay University Saint‐Cloud France; ^2^ Department of Clinical Hematology and Cellular Therapy, Saint‐Antoine Hospital, AP‐HP Sorbonne University Paris France; ^3^ Université des patients Sorbonne University Paris France; ^4^ IRIS 997 INSERM Aubervilliers France; ^5^ Département de Médecine Gustave Roussy Villejuif France; ^6^ INSERM, IRD, SESSTIM, Sciences Economiques & Sociales de la Santé & Traitement de l'Information Médicale, ISSPAM Aix Marseille University Marseille France; ^7^ Université Paris Est Créteil Creteil France

**Keywords:** care pathway, co‐construction, colorectal cancer, participatory research, patient partners, professionals' perceptions

## Abstract

**Introduction:**

Qualitative research on the perceptions of healthcare professionals involved in cancer care about their respective roles in the patient care pathway is limited. Therefore, the aim of this qualitative study was to document these perceptions.

**Methods:**

A multidisciplinary team that included patient researchers constructed a semi‐structured interview guide on the perceptions of the colorectal cancer care pathway by professionals. Interviews were conducted with healthcare professionals from two French hospitals that manage patients with colorectal cancer. Then, the interviews were fully transcribed and analysed by the whole multidisciplinary team.

**Results:**

Thirteen healthcare professionals were interviewed (six nurses, four physicians, one psychologist, one social worker and one secretary). They described the colorectal care pathway using a great lexical diversity and listed a significant number of professionals as taking part in this pathway. Among the people mentioned were healthcare professionals working inside and outside the hospital, family members and non‐conventional medicine practitioners. However, they did not spontaneously mention the patient. Their views on the role of the referring physician, the general practitioner and the patient were further explored. The interviews highlighted the coordination difficulties among the various professionals, particularly between general practitioners and hospital teams. These data provided interesting elements for developing a tool to help coordination among professionals.

**Conclusions:**

This preliminary study, with its participatory design, brings interesting elements of reflection on the care pathway for patients with colorectal cancer. It will continue through the creation of a larger participatory project.

**Patient or Public Contribution:**

Patient partners were included in all steps of this study. This transdisciplinary project was coordinated by a group composed of three patient partners, two healthcare professionals and two humanities and social sciences researchers. Their knowledge of the patient's perspective on the care pathway enriched discussions from the study design to results analysis.

## 1 Introduction

Scientific advances have allowed moving part of the management of patients with cancer to outpatient settings. In this context, the creation of a personalized and coordinated pathway to ensure quality care for all was one of the objectives of the 2009–2013 French National Cancer Plan [1] and also of the American Cancer Society [2]. However, French data indicate that 34% of patients did not have a coordinated care pathway. These data also revealed the heterogeneity of resources and practices among cancer centres, particularly concerning the coordination between city and hospital [3], despite the regional cancer network contribution to care organization [4]. Indeed, the development of outpatient management relies not only on the role of different healthcare professionals (e.g., general practitioners [GPs], nurses and pharmacists) [5] but also on the commitment of patients, informal caregivers and patient associations [6, 7, 8]. Colorectal cancer (CRC) is the fourth most common and the fifth most deadly cancer worldwide [9]. In France, CRC is the second cause of mortality, and its incidence is high in both sexes [10]. CRC management is an example of care pathway in which multiple actors are involved in the different steps: screening, diagnosis, announcement, treatments (e.g., surgery, radiotherapy and chemotherapy) and recovery.

The issues raised by the care pathway have been particularly studied in the screening and post‐cancer phases. Different authors have highlighted suboptimal understanding of information, inadequate communication and poor relationships with healthcare professionals as barriers to CRC screening participation [11, 12]. Several studies have highlighted the difficulties encountered by GPs in assuming their roles in cancer prevention and care due to organizational issues, insufficient training and problems in communicating with the hospital teams [13, 14, 15]. In addition, they have reported the patients' dissatisfaction concerning the CRC diagnosis announcement and the quality of the information they received [16].

Lastly, concerning recovery, many studies have described important chronic adverse events linked to CRC treatment that negatively affect the patients' quality of life and return to work [17, 18].

Several studies have explored patients' views throughout the care pathway. The high rate of patients recognizing the need for better interaction with the healthcare system is a clear message in favour of improvement. The need for coordination between healthcare professionals also seems essential in these studies [19, 20]. To complete this patient‐centred vision, we thought it would be interesting to look at the healthcare professionals involved in the treatment phase. We hypothesized that the identification of the different healthcare professionals and their respective roles by actors involved in the CRC care pathway varies according to the actors and their place in the different steps of the CRC care pathway. These variations may negatively affect care coordination and also the patients and their informal caregivers' experiences of cancer management. Therefore, the main objective of our study was to explore the perceptions of the different professionals of the CRC care pathway at hospital about their own roles and the roles of the other stakeholders. Secondary objectives were to describe the coordination modalities of this pathway and the obstacles perceived by the professionals and to collect their suggestions for improving the CRC care pathway. Finally, as this project was born from the outset of cooperation between clinicians, patient partners and researchers, we would like to discuss the contribution of this approach to a research project on care pathways and consider this approach as one of the secondary objectives of the study.

## 2 Methodology

Involving patients and citizens has become a new modality of partnership in research [21]. Some authors have described the different modalities of their involvement in cancer research [22], and several studies have demonstrated the interest and benefits of their participation in clinical [23] and interventional research [24, 25]. Therefore, patient partners (as they chose to be named) were included in all steps of this study. Specifically, the project was coordinated by a group composed of three patient partners, two healthcare professionals (an oncology resident and a clinical psychologist also sociologist), a researcher in medical ethics and sociology of health and an engineer in public health research. This transdisciplinary approach, in which knowledge from experts in different fields and knowledge from non‐academic stakeholders were combined, was affirmed throughout the project.

This qualitative study was based on semi‐structured interviews with healthcare professionals working at two cancer care centres in Paris: one from a university hospital and the other from a comprehensive cancer centre. The selection of professionals was carried out by the partner patients using a list of professionals considered fundamental in the CRC care pathway: oncologists, surgeons, anaesthetists, nurses, psychologists and social workers. The interview guide (Supporting Information S1: Table 1) was designed in several steps: The partner patients wrote the first version that was discussed in two steps with the other group members. The questions were designed to explore the different actors' perceptions of the CRC care pathway, of their own roles and of the other stakeholders' roles. The interviews were conducted by a public health research engineer after training with the patient partners, the psychologist and the medical ethics researcher.

Professionals were contacted via email and telephone. Due to various COVID‐19 pandemic–related restrictions, interviews were conducted via videoconference (March 2021–February 2022). Interview data were analysed using the thematic analysis method [26, 27, 28]. After recording and transcription, each interview was independently coded by a researcher–patient partner pair and harmonized by comparing the two coding versions. The whole team discussed the results of the coding and organized the codes into themes (Supporting Information S1: Tables 2 and 3). This analysis allowed the listing of the stakeholders identified by the interviewed professionals and their respective roles and to determine the interviewees' perceptions of the CRC care pathway (including steps, organization, coordination, obstacles and suggestions). Figure 2 was created using Gephi 0.10.1 software. This qualitative study follows the Standards for Reporting Qualitative Research (SRQS) guidelines [29].

## 3 Results

### 3.1 Participants

Thirteen professionals from the two cancer centres gave their consent to participate in the study: six nurses, four physicians, one psychologist, one social worker and one secretary. Their median age was 44 years (range 28–57). To avoid the risk of identifying the interviewed professionals, Table 1 does not list their gender, exact age and place of work.

**Table 1:** Professionals interviewed (age group).

Medical oncologist (30–39 years)
Advanced practice nurse (30–39 years)
Consultation nurse (20–29 years)
Consultation nurse (50–59 years)
Psychologist (20–29 years)
Medical oncologist (50–59 years)
Nurse coordinator (40–49 years)
Medical oncologist (30–39 years)
Announcement and consultation nurse in digestive surgery (50–59 years)
Digestive surgeon (50–59 years)
Announcement and consultation nurse (50–59 years)
Medical secretary (50–59 years)
Social worker (30–39 years)

### 3.2 The Diversity of Stakeholders in the CRC Care Pathway

**Figure 1:**
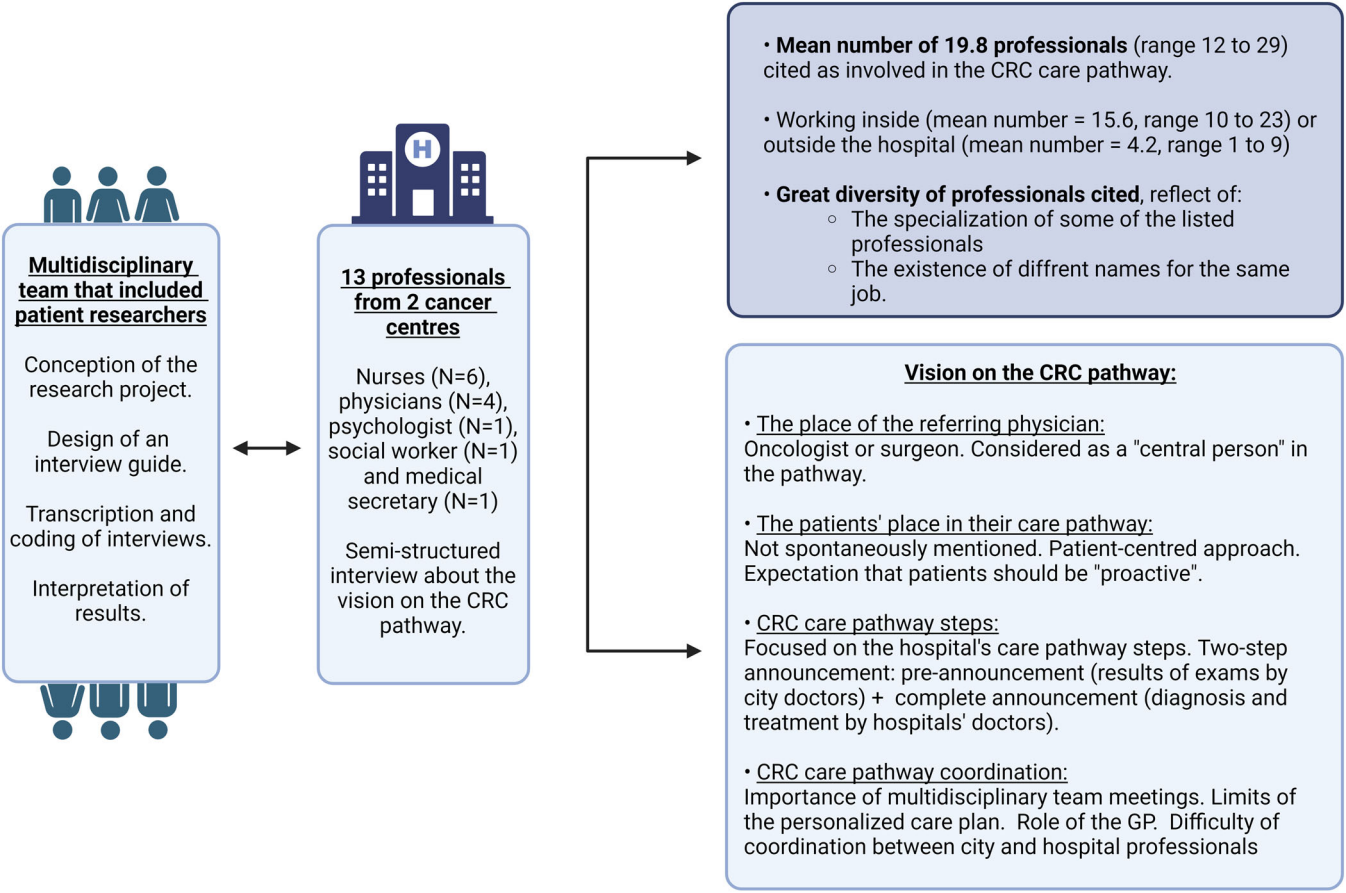
Design and the main results of the study. CRC, colorectal cancer.

**Figure 2:**
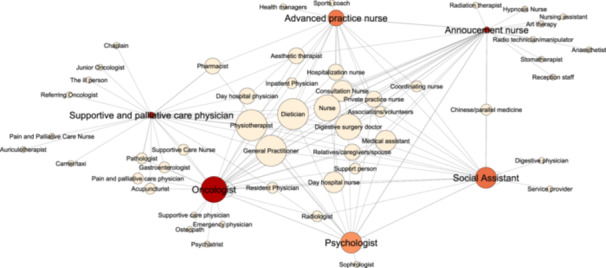
Network of the people mentioned as implicated in the CRC care pathway by professionals interviewed at the two cancer centres. The dot size is proportional to the number of times each individual was cited. Red dots correspond to the interviewed individuals. The red colour intensity is proportional to the number of professionals mentioned by that interviewee.

First, we noted a great diversity of professionals listed by the interviewees as involved in the CRC care pathway (Table 2). This reflected the specialization of some of the listed professionals (e.g., ‘coordinating nurse’ and ‘supportive care nurse’) and also the job name heterogeneity between hospitals. Sometimes, interviewees working in the same hospital used different terms to refer to the same professional (e.g., ‘supportive care nurse’ or ‘pain and palliative care nurse’). Moreover, they designed some professionals once by their function and then again by their rank (e.g., ‘senior physician’, ‘oncologist’ and ‘day hospital physician’). We identified 76 different terminologies for professionals in the CRC care pathway. By taking this heterogeneity into account, we calculated that the interviewees mentioned a mean number of 19.8 professionals (range 12–29) involved in the CRC care pathway. Most of them work in hospitals (Figure 1). The most frequently cited professionals were oncologists and GPs (12/13), followed by nurses (11/13), surgeons (11/13), dietitians (10/13) and psychologists (10/13) (Table 2). Some of the mentioned professionals practiced alternative medicine, mainly in the field of supportive care (e.g., auriculotherapy practitioner, osteopath and fire cutter). Moreover, professionals working in the hospital, but not directly as caregivers (e.g., medical secretary or clinical research officer), were also mentioned by the interviewees. Lastly, the family and relatives were mentioned as stakeholders in the CRC care pathway: relatives (5/13), family (4/13) and children (4/13). The number of stakeholders and the type of mentioned professionals varied greatly among interviewees, as illustrated in Figure 2, and between centres. Oncologists and coordinating nurses were the interviewees who gave the longest lists of professionals implicated in the CRC care pathway.

**Table 2:** Members of the care pathway mentioned by the interviewees.

Named member	Number of hits^a^	Named member	Number of hits^a^
Oncologist	12	Consultation nurse	2
Attending physician	12	Supportive care nurse	2
Nurse	11	Medical specialist	2
Digestive surgery doctor	11	Chief medical officer	2
Dietitian	10	Pain and palliative care physician	2
Psychologist	10	Outpatient physician	2
Social assistant	9	Geriatrician	2
Physiotherapist	9	Senior physician	2
Announcement nurse	8	Hospital agent	2
Day hospital nurse	7	Service provider	2
Gastroenterologist	7	Spouse	2
Resident physician	7	Associations/volunteers	2
Radiologist	6	Support and coordination nurse	1
Healthcare aide	6	Surgical nurse	1
Aesthetic therapist	6	Pain and palliative care nurse	1
Coordinating nurse	5	Fast‐track nurse	1
Hospitalization nurse	5	Hypnosis nurse	1
Liberal nurse	5	Dressing nurse	1
Acupuncturist	5	Care nurse	1
Day hospital doctor	5	Cancer physician	1
Pharmacist	5	Cardiologist	1
Relatives	5	Support and palliative care physician	1
Advanced practice nurse	4	Supportive care physician	1
Stomatherapist	4	Digestive doctor	1
Anaesthesiologist	4	Junior oncologist	1
Psychiatrist	4	Homeopathic physician	1
Medical assistant	4	Dermatologist	1
Family	4	Physician	1
Children	4	Clinical research associate	1
Person of trust	4	Reception staff	1
Pathologist	3	Sophrologist	1
Hospitalization doctor	3	Cleaner	1
Referring oncologist	3	Osteopath	1
Radiation therapist	3	Auriculotherapy practitioner	1
Emergency department physician	3	Fire cutter	1
Healthcare executive	3	Sexologist	1
Medical secretary	3	Radiotherapy technician	1
Sports coach	3	Chaplain	1

^a^ Each number represents how many among the 13 interviewed people mentioned a specific professional as implicated in the CRC care pathway pathway.

### 3.3 The Place of the ‘Referring Physician’ in the CRC Care Pathway

The ‘referring physician’ could be either the oncologist or the surgeon, in function of the patient's care pathway and treatments. All interviewed people considered the referring oncologist as ‘the central person’, ‘a kind of conductor’ and ‘the common thread in the care process’. However, they also mentioned that sometimes, the oncologist would only meet the patient at the beginning and the end of the ‘active’ treatment process for the reassessment phase. Among participants, some oncologists suggested that the patient should be able to contact them throughout the care pathway (Supplementary Information S1: Table 3).

Throughout their care pathway, patients also met other oncologists who worked in the day hospital and resident doctors. To compensate for the low number of visits by the referring oncologist, one of the interviewees suggested the possibility of pairing the referring oncologist with a junior oncologist, who would have more time to be there for the follow‐up consultations:

I think one important thing is maybe companioning a senior oncologist who does the treatment changes, who is the referring physician, etc. and a junior oncologist, but one who would be fixed and that they [the patients] would really see regularly.

(Prof 6)

When no chemotherapy was needed for CRC management, the referring physician could also be the surgeon who was then responsible for the patient's follow‐up. In one of the two centres, the surgeon was systematically accompanied by nurses who were present at all consultations and were considered the referring healthcare professionals, in addition to the surgeon. For patients who required surgery and then chemotherapy, both the surgeon and the oncologist were the referring physicians who alternatively handled the follow‐up.

### 3.4 The Patients' Place in Their Care Pathway

Interestingly, none of the interviewees spontaneously mentioned the patient among the care pathway actors. They did this after dedicated questions. Nevertheless, an interviewee described a personal vision of the patient‐centred approach:

Their place is central in fact. Patients are in the middle and then all the others revolve and gravitate around them. Patients are in the middle; they are the ones we're looking out for and everything that happens around them is for them.

(Prof 10)

Others have a strong expectation that patients should be ‘proactive’. This expectation was expressed using a variety of terms: ‘active’, ‘major actor’ and ‘responsible’. Some interviewees also emphasized that patients have duties and responsibilities, such as sharing what they expect from the treatment, understanding the information and even checking whether the steps are done properly and ‘correct our mistakes’. Some interviewees also highlighted the fact that some patients may desire to receive information and to participate in making decisions affecting them (shared decision‐making model). Conversely, other patients may prefer not to be fully involved in their care pathway and maintain a certain ignorance about it:

… it is both a shared decision, but it is also shared in the sense that if the patient does not wish to be an actor but wishes to be carried between quotation marks by the team, this must be heard. Finally, it is something that must be accepted or something that is like that, is shared, that can be discussed, understood and as always, individualized with each patient.

(Prof 6)

We noted the absence of any reference to therapeutic education, decision aids or information support for therapeutic choice in the interviewees' speeches.

### 3.5 Different Representations of the CRC Care Pathway Steps

Depending on the role of the interviewed person, the CRC care pathway description was more or less extensive. The care pathway steps mentioned in the interviews were as follows: diagnosis period, referral to the appropriate service, multidisciplinary team meeting, consultation to announce the diagnosis and the therapeutic project, active treatment (chemotherapy, radiotherapy, surgery or a combination), supportive and palliative care and monitoring/re‐evaluation.

The interviewees who listed fewer steps generally did not mention supportive care, palliative care, monitoring and reassessment. Notably, interviewees did not mention complications as if the care pathway was linear. Furthermore, they focused on the care pathway steps that take place in the hospital and did not mention the ambulatory care provided by private‐sector professionals or outside‐hospital care networks.

Similarly, they did not specify the precise end of the care pathway. Conversely, they talked mainly about the beginning of the care pathway, the diagnosis and its announcement. They often divided the announcement into two stages: a pre‐announcement by the healthcare professional who made the diagnosis (often the gastroenterologist), followed by a more complete announcement with the cancer diagnosis delivery by the oncologist or the surgeon to explain the disease and its treatment:

In fact, the announcement is not necessarily done by our team because we are in the surgical field. The announcement is done more frequently by the primary care physician or by the gastroenterologist, because it is after the colonoscopy that we know whether it is cancer or not.

(Prof 11)

There was an expectation of pre‐announcement on the part of the professionals who would subsequently intervene to ‘prepare’ the patient for the diagnosis. As no professional practicing outside the hospital was interviewed, it was not possible to collect the views of the professionals who carry out these pre‐announcements. Some interviewees suggested that the pre‐announcement step may be carried out in inappropriate conditions (e.g., in the corridor) or without sufficient information about the next steps of the care pathway:

An announcement without explaining what the treatment is going to be and the solutions we can provide, it's complicated … .

(Prof 1)

They also pointed out the fragmentation of the information given at the time of the announcement or its repetition during the subsequent consultations, particularly with coordination and/or announcement nurses. Therefore, they described the announcement as a multistep and multidisciplinary process:

There is the announcement by the doctor, then by the nurse; it is good that the nurse can give again the information and check whether the information given during the first consultation has been understood. And to say things again in different words. The fact that there are several people involved means that there are different modes of communication and therefore, the patient can be pushed to understand and re‐questioned, re‐challenged.

(Prof 1)

### 3.6 The Supportive Care Place in the CRC Care Pathway

Supportive care was not mentioned in the same way by all interviewees and at the two cancer centres, which are organized differently. Supportive care was mentioned 26 times by interviewees who worked in the centre with a dedicated supportive care team directly linked to the oncology department and eight times by interviewees in the centre without supportive care team. This difference could also be explained by the fact that at the first centre, one of the interviewees was a member of the supportive and palliative care service. The supportive care place ranged from its integration throughout the whole care pathway to a request based on the patient's needs. Some interviewees identified supportive care as particularly crucial for patients with advanced cancer. Others stressed the importance of supportive care, including physical activity, before any treatment (e.g., before surgery).

### 3.7 The CRC Care Pathway Coordination

We were interested in identifying the tools of coordination mentioned by the interviewed professionals. Multidisciplinary team meetings are an important tool in the management of patients with cancer. Their aim is ‘to discuss the patients’ diagnosis and conditions and organize their treatment plan according to the most appropriate evidence‐based protocols' [30]. Surprisingly, only two of all interviewed professionals mentioned multidisciplinary team meetings. Some interviewees also mentioned the personalized care plan, which is also called the treatment plan [31], intended for patient information. They considered it as a real tool for coordination among healthcare professionals. This document, in paper format and the content of which varies from hospital to hospital, was associated with the coordinating nurses' work. Some interviewees cited its sometimes‐limited accessibility in the patient's medical file or its difficulty updating as one of the reasons to explain some coordination difficulties among healthcare professionals:

the personalized care plan, but unfortunately, it's not used enough or not visible enough in the records.

(Prof 2)

Several professionals mentioned the digitization of this document as a possible improvement. This could make the document more easily shareable and adaptable in function of the changes in the patient's care.

So yes, digital tools, applications, things that are a little bit fun, not big pamphlets, where you give a sheet of information on both sides written in a very small format, with a whole bunch of information, some very important, very relevant, other much more blah‐blah. So, there are things that are perhaps a little more fun.

(Prof 8)

The third “tool” was the GP itself, who was the most cited out‐of‐hospital healthcare professional. The GP was seen as the recipient of medical correspondence and the personalized care plan document:

… I know that all reports are sent to the general practitioner and then there's also a part of the primary care management, in terms of some of the prescriptions, but also tracking the overall health of their patients.

(Prof 5)

Interviewees attributed the following roles to the GP: the eventual pre‐announcement, management of the cancer treatment–related adverse events and coordination of outpatient care. This coordination sometimes remained difficult because not all city healthcare professionals (e.g., home nurses) had access to the patient's file. The GP's role in screening or participating in the cancer therapeutic decision‐making was not mentioned. Some interviewees highlighted communication difficulties with the GPs and suggested that GPs may lack time, may be unable to reach the hospital oncology teams and may not be comfortable with some cancer‐related situations:

I have worked in places where we could really say that we had a real task delegation; however, there also primary care physicians who finally fade away a bit. We don't tell them much, we don't take care of them. And finally, patients sometimes feel that they don't even have to be in the loop.

(Prof 6)

## 4 Discussion

The aim of this qualitative study was to explore the perceptions on the CRC care pathway by interviewing the implicated healthcare professionals. Although exploratory, this study identified some of the specific features of this care pathway and the necessity of improving the coordination among its different actors. The interviewed professionals had different views on the definition of the CRC care pathway that could be summarized as follows: one that is patient‐centred and adapted to each clinical situation and another where the care pathway is thought as a means of organizing care at the institutional level. The perception of the different care pathway steps was heterogenous, particularly when the care pathway starts (screening or diagnosis) and ends (after chemotherapy, monitoring or remission). These differences varied in the function of the interviewed professionals. Surprisingly, some steps were never mentioned (e.g., possible side effects or end of life [32]) and others (e.g., out‐of‐hospital steps and surveillance phase) were mentioned only a few times. Some steps were extensively described and discussed, particularly the diagnostic work‐up and the possible pre‐announcement (i.e., information about the diagnosis given before the patient is referred to the cancer centre). In France, the conditions of the diagnosis and treatment announcement have been very well established with a diagnosis disclosure procedure [33]. However, our work underlines that there is often a pre‐announcement time, sometimes performed in inappropriate conditions. The fluidity of the transition and continuity between this pre‐announcement time and the beginning of cancer treatment depends on the coordination between the outside‐hospital professionals and the hospital team. Our study underlines some issues concerning this coordination, particularly with the GP. These coordination difficulties could explain why the interviewees' vision of the CRC care pathway was very hospital‐centred, except for the diagnosis step. There was a discrepancy between the recommendations concerning the GP's place in the care pathway and the interviewees' views. In France, the GP is expected to be implicated at each step: ‘The increased involvement of the GP, before, during and after the treatment of their patients with cancer, is a major focus of successive cancer plans’ [34]. Nevertheless, in our study, the GP's role in relation to the hospital CRC care team and the patient was not precisely identified. Some interviewees also mentioned that some patients did not want their GP to be included at all in the coordination loop. Those results are consistence with previous study conducted on patients' views on GP's role in cancer care pathway [35]. It is worth noting that the outpatient cancer care networks, which should facilitate the professionals' coordination for outpatient care, were not mentioned.

Concerning coordination, interviewees underlined the limits of the existing tools. The personalized care plan document, which should be an information support for patients, was seen as a coordination tool. However, in the interviewees' opinion, it presented some limitations: its impossibility to be changed following the occurrence of specific events in the patient's care, its inaccessibility to outside‐hospital professionals and its content heterogeneity (the included information is different at each hospital).

A tool specifically dedicated to coordinating the patient's care pathway seems interesting and should make it possible to improve it [36]. This tool should be accessible to all involved professionals, ideally in digital format, and provide a clear identification of who to contact in each situation. However, it raises questions about data security, accessibility by healthcare professionals not working in the hospital and also by patients. It also brings the question of whether all patients and professionals are able to use digital tools [37]. In view of the semantic diversity observed in our study, the implementation of this coordination tool would require upstream work to harmonize the used terminology and at least the healthcare professional nomenclature, to make it easier for everyone to identify the role of the different professionals and to promote collaboration.

Finally, our work raises the question of the role of patients who were rarely spontaneously mentioned among the actors of the CRC care pathway. Exploring the interviewees' perception of the patients' role, we noted the ambivalence in the interpretation of the patient‐centred care pathway. On the one hand, some expect patients to be compliant and to follow the medical team's suggestions as strictly as possible. On the other hand, others expect patients to fully understand information and to become autonomous, proactive and responsible.

Yet, more recent definitions specify that patient‐centred care is about focusing care on the needs of each individual. This implies ensuring that clinical decision‐making is guided by the patients' preferences, needs and values, and that the provided care is respectful of and responsive to the patients. This diversity of the patient‐centred care interpretation was already described in the scoping review by Pel, Engelberts, and Schermer [34]. In recent years, a dynamic transition has been underway to propose a vision of a patient alongside the healthcare professional circle with a participatory role [38]. In this way, patients are not sidelined and can have a coordinating role in their care. However, this requires ethical vigilance regarding the patients' ability to take on this role, their availability and means to do so, the transmission of information and the necessary support and accompaniment if and when needed.

Several previous studies already reported the patients' views about the CRC pathway. They were summarized by Kotronoulas et al. [19]. They reported that half of the needs were related to information provision and patient–clinician communication. Also, emotional support and reassurance (especially when dealing with fear of cancer recurrence), more information, better patient education and better interaction with the healthcare system were the most prominent needs overall. The authors strengthen that ‘better coordination among healthcare professionals also is key, especially as patients transition from acute to rehabilitation care’.

Our study was original in that it brought the viewpoint of professionals involved in the CRC care pathway, which is little reported in the literature. It is interesting to note that some of the needs expressed by patients in dedicated studies were also expressed by professionals in our work, particularly in terms of information delivery and coordination between caregivers. As only two cancer centres were included in this exploratory study and only a few professionals were interviewed due to the COVID‐19 pandemic–related limitations, no generalization can be drawn from this study. The lack of participation of professionals involved in outpatient care also restricted the analysis of the CRC recovery step and its specific issues.

However, our study highlights some areas of work on the care pathway that need to be explored. These exploratory findings have been used as the basis to design a large multicentre research project on how the CRC care pathway can be improved with a participatory approach, which is currently underway (4P project, financed by French Cancer Institute INCa No. 2022‐059).

Our study is also a good illustration of the feasibility and the contribution of a research project entirely based on participatory research. This study was totally co‐constructed with patients who encounter cancer (i.e., patient partners, here) who obtained a specialized diploma at the French patients' university [39]. This project mobilized their experiential knowledge of the disease [40], although they did not have CRC. This allowed them to keep a critical distance from the research topic. Their knowledge of the patient's perspective on the care pathway enriched the discussions from the study design to the analysis of the results. In this participatory project, we encountered several obstacles to the participation of patient partners, as described in the literature [41, 42]. Indeed, patient and public involvement is not current in research projects, and some challenges need to be overcome, notably methodology training, financial compensation and social recognition of their role in research. The patients' experiential knowledge is considered complementary to that of healthcare professionals for improving the healthcare system, including the cancer care pathways [22, 23, 25, 40, 43, 44].

## 5 Conclusions

This exploratory study opens up some interesting avenues for improving the CRC care pathway. Indeed, each professional seems to be focused on his or her function and has little regard for the actions of other professionals in the care pathway. This observation is probably not specific to France and could be encountered in other countries, as has been shown in the case of GPs in relation to prevention. Improving the care pathway for patients will require better visibility of its different steps and therefore of the involved professional. To improve visibility for patients and professional coordination (for in and outpatient care), we suggest that the terminology used to designate healthcare professionals should be harmonized. A tool, probably digital, should be proposed to provide an overall view of the care pathway and the roles of each professional. Our study also demonstrates the feasibility and value of involving patient partners at every stage of a research project.

## Author Contributions

**Matthieu Delaye:** conceptualization, funding acquisition, project administration, methodology, formal analysis, writing–original draft, writing–review and editing, visualization, data curation, software. **Alice Polomeni:** conceptualization, funding acquisition, methodology, formal analysis, supervision, writing–review and editing, visualization, validation, data curation. **Sylvie Faiderbe:** conceptualization, methodology, formal analysis, visualization, data curation, writing–review and editing. **Nadège Berlioz:** conceptualization, methodology, visualization, data curation, writing–review and editing. **Chaïma Benssekoum:** methodology, investigation, data curation, formal analysis, visualization, writing–review and editing. **Aude Guillemin:** investigation, formal analysis, writing–review and editing. **Thomas Pudlarz:** investigation, formal analysis, writing–review and editing. **Sandrine de Montgolfier:** conceptualization, funding acquisition, project administration, methodology, formal analysis, supervision, writing–review and editing, visualization, validation, resources, data curation, software.

## Ethics Statement

The study was approved by the INSERM Ethics Evaluation Committee (IRB00003888/INSERM No. 21‐831 of 6/07/2021).

## Conflicts of Interest

The authors declare no conflicts of interest.

## Supporting information

Supporting information.

## Data Availability

The final data set is completely pseudonymized. These data may be provided to researchers running other research studies. Future research would be compatible with this research project. Participant information document reflects future use of these data in research.
