# Supplementary material for: Professionals' Perceptions of the Colorectal Cancer Pathway: Results of a Co‐Constructed Qualitative Study
Source: Health Expect. 2024 Jul 14;27(4):e14146. doi: 10.1111/hex.14146 (PMC11246595; doi:10.1111/hex.14146)
Supplement: Supplementary file 1 — Supporting information. [file HEX-27-e14146-s001.docx]

**Supplementary Table 1: Semi-structured interview guides on the care pathway**

| **Socio-demographic data** |
| --- |
| Age |
| Gender |
| Job / activity |

| **Diagnostic** |
| --- |
| What are the ideal conditions for diagnosing and announcing colorectal cancer (systematic screening? symptoms? Consultation? In your opinion, which doctor should make the announcement (general practitioner, radiologist, oncologist)? Which healthcare professionals should be involved in the announcement? |

| **Care pathways** |
| --- |
| How would you define a care pathway? What are its main stages?  In your opinion, is the entire team (medical and paramedical) aware of the patient's care pathway?  Is a document detailing the patient's personalized care path systematically provided? |
| What is the care pathway for colorectal cancer patients? What is the care pathway for the patient you support? |
| Who are the key players in this care pathway?  Do these players intervene systematically, according to the patient's situation, at his or her request, or at the request of a healthcare professional?  At what points in the process do you become involved? |
| How does care coordination work between the various care providers (intra-hospital and hospital/city)?  Do you have the impression that there are collaboration difficulties between certain professionals?  for what reason(s)? |
| Which steps go best? And which are the most difficult (a) for you and (b) for the patient? |
| What roles do other healthcare professionals play at each of these stages? [What other care providers are needed?] |
| What needs do you see for improving the care pathway (a) from your point of view and (b) from the patient's point of view? |
| What role/place do you see for the patient in the care pathway? |
| What is your view of the care pathway and the roles of the various players (healthcare professionals and caregivers)?  Is there room for patient or former patient associations? |

**Supplementary Tables 2: List of the themes**

**The stages of the care pathway**

1. The diagnostic process
   1. The different ways of entering the disease
   2. Initial announcement, an often complicated step
2. The conditions for the announcement at the hospital:
   1. Variability (duration of consultation, location, professionals involved, presence of a companion, resumption of the condition of announcement by the nurse)
3. Variability in the treatment phases in CRC
   1. Types of pathways according to the cancer (localized or metastatic diseases
   2. Types of treatment involving different professionals
   3. Materials to present them to patients
4. Post-treatment follow-up: regularity, monitoring assessments; Delays between check-ups
5. Supportive care, great variability in the terms of the proposal
6. The professionals involved and their roles
7. Materials to follow the care pathway

**The role of the patient as seen by professionals:**

1. Expectations of professionals sometimes conflicting
2. the modalities of adapting the caregiver-patient relationship
3. The roles of the entourage

**Difficulties encountered in the care pathway:**

1. Organizational problems (duration of consultations, lack of coordination staff, relations within the department, inter-service relations, links between the cities and hospitals, recognition of the time devoted to the organization)
2. Consequences of these problems for the patient (variability in the information transmitted, dehumanization of the doctor-patient relationship, difficulty in implementing shared medical decision-making)

**Suggestions for improving the care pathway**

1. Propose a referral professional (alone or in pairs)
2. Have dedicated coordination professionals
3. Improving the city-hospital relationship (hotline, shared city-hospital file, coordination time for city professionals)
4. Creation of a pathway tracking tool (easy to update; for professional patients; listing professionals and their roles)

**Supplementary Table 3: extract from several supporting quotes**

| **Theme/Sub-theme** | **Code** | **Example quote** |
| --- | --- | --- |
| **The stages of the care pathway** | | |
| The diagnostic process | The different ways of entering the disease | *"I think that already right off the bat it's good if the gateway was the primary care physician, that they have an initial preparation on their part if they find something concerning and I think you have to be transparent with patients and say here I am referring you [to the hospital], because I found something concerning."* |
|  |  | *"In fact, the announcement is not necessarily made at hospital, since we are in the surgical field. The announcement is made more frequently with the attending physician" "or with the gastroenterologist, since it is following a colonoscopy that we know if it is cancer or not".* |
|  | Initial announcement, an often complicated step | *"An announcement without explaining what the treatment behind it is going to be and the solutions we can provide, it's complicated..."* |
|  |  | *"The gastroenterologists who do the colonoscopies, they string them together eh you know how it is and it's true that there are announcements that remain quite brutal."* |
|  |  | *"There are still quite a few patients who tell me about the announcement between two doors. The doctor has to say, say what it is, that is to say, say the word cancer because there are still patients who arrive... The word has not been pronounced, the doctor has avoided it and the patient does not know what he has. That's not possible anymore.”* |
| The conditions for the announcement at the hospital: | Spreading the announcement over several consultations | *"There is the announcement by the doctor, then by the nurse, it is good that the nurse can also re-challenge the information, the understanding of the information given during the first consultation. And to say things again in different words. The fact that there are several people involved means that there are different modes of communication and therefore the patient can be pushed to understand and therefore re-questioned, re-challenged (...) Each time we meet the patient, we all have a little challenge in trying to assess whether they know what is going on.* |
|  |  | *"A first [announcement] to explain the diagnosis, (...), the course of treatment, what can be proposed to him, as care, as treatment", then a "time of reflection which is surely one week, two weeks ideally" (...), and "a second consultation of medical announcement, where the patient will have the time to reflect, to settle down, to understand also what happens to him. (...) and to see the oncologist again (...) to see if everything has been understood, and to begin to initiate treatment.* |
|  |  | *"What we do is see them again before their systematic treatment, when they come to the outpatient clinic. So, often, it's been one to two weeks after the medical announcement. More or less. It depends, actually, if it's urgent or not. And when they are hospitalized, the patients are also seen. But we'll say in a way ... sometimes we have a little more time, so we don't necessarily see them right away when they are taken in.* |
| Variability in the treatment phases in CRC | Multidisciplinary team meetings | *"He [the doctor] will discuss his file in a multidisciplinary meeting, this famous consultation meeting to see what treatment really needs to be put in place, surgical or not, whether to do per or postoperative chemotherapy?"* |
| Supportive care, great variability in the terms of the proposal | Supportive care in advanced cancer | *"Of course, in the pathway of the metastatic patient, we haven't talked about supportive care, palliative care, but we're going to integrate it earlier, knowing that we're already with someone who is metastatic. And so the idea of integrating supportive care very early on seems fundamental to me. So we do it as a team. At the beginning of the treatment, we try to put the dietician in place. The team of doctors and nurses for pain and palliative care and other professions, if necessary. Calling on other supportive care scales seems very important to me."* |
|  |  | *"I think the stages that are best supported by the patient is anything that is managed in supportive care, because it's still a time when the care is less aggressive and we talk about quality of life."* |
|  |  | *"And then we have a slightly different experimentation for patients who come in at a more severe stage, for example, with metastases already present at diagnosis. And there, we're trying to get supportive care in very early."* |
|  | Supportive care before surgery | *"(Pre-habilitation) is getting patients to do exercises to prepare them for surgery. Patients who have cancer. Physiotherapists, patients have difficulty finding them in town and if we had a structure that was like a gym, where we could have people who would help them prepare for surgery through physical activity, that would be important.”* |
| The professionals involved and their roles | Central role of the referent physician | *"a central person, the oncologist" "[he] is a kind of conductor”* |
|  |  | *"We [the referent physician] are really the common thread of care."* |
|  |  | *"We [the referent physician] are the ones who explain the project and organize the pace of treatment follow-up."* |
|  | Other oncologists are met along the care pathway | *"There is the day hospital physician who monitors treatment tolerance before each chemotherapy injection."* |
|  |  | *"There is the hospitalization physician who takes care of complications during hospitalizations"* |
|  |  | *"They can call me- but otherwise I [the referent physician] only see them again at re-evaluation."* |
|  | Role of the general practitioner (GP) | *"...In the middle of it all, there's the primary care physician. But that's more for the relay with the city and the house."* |
|  |  | *(...) the general practitioner was really a fundamental player. I have worked in places where we could really say that we had a real delegation, whereas there are attending physicians who finally fade away a bit. We don't tell them much, we don't take care of them. And finally, patients sometimes feel that they don't even have to be in the loop. It is often the patient who chooses. In some territories, the attending physician is really an attentive physician. In short, I won't use any qualifiers. Here, we really have a link with the attending physician and that seemed fundamental to me."* |
|  |  | *"...I know that all the reports are sent to the general practitioner and then there's also a part of the primary care physician's management, in terms of some of the prescriptions, but also tracking the overall health of their patients."* |
|  |  | *"...We know obviously the general practitioner is still made aware of what has been decided and is always in contact to be able to advise the physician of what has been found, what has been confirmed and the conducts to be followed and even the aftermath of this management concerning the patient in question."* |
| Materials to follow the care pathway | Care Personalized plan(PPS) | *"(...) in the end, I give them very few documents other than the PPS because they are already drowning in papers.* |
|  |  | *"Normally, that exists is the personalized care plans, but unfortunately, it's not used enough or not visible enough in the records."* |
|  |  | *"Yes, absolutely. Here (…), it's the coordination nurses who do it, but indeed, he is given a personalized care plan with all the phone numbers that can be useful."* |
|  |  | *"I think the supports are important, yes. So let it be tailored. Like I said, I think giving everything at once, giving everything to the patient at once, I think that's counterproductive because there's too much."* |
| **The role of the patient as seen by professionals:** | | |
| Expectations of professionals sometimes conflicting  parfois opposés | A central place but without any particular expectations | *"His place is central in fact. He is the middle and then all the others revolve around and gravitate around. He's in the middle, he's the one we're looking out for and that everything that happens around him is for him."* |
|  | Ambitious expectations | *"I think that his role is to ask questions in order to understand (...) to master the information a little and therefore to be able to control that things are well done. (...) to tell us to express what he feels (...) to be really questioning, to understand and to see to be able to help us and to correct sometimes our mistakes".* |
|  |  | *"They ask questions, they want to know, so they are active in their care."* |
|  |  | *"I think patients they have a great responsibility and duties in their care journey."* |
|  | A position to be adapted according to the patients | *"It's very variable from one patient to another. For me, they should all have, always with these models of shared medical decisions, this place that must be given to them as actors in their care, but which at the same time is not necessarily mandatory. That is to say that for me with this model it is both a shared decision but it is also shared in the sense that if the patient does not wish to be an actor but wishes to be carried between quotation marks by the team, this must be heard and that finally, it is something that must be accepted or something that like that, is shared, that can be discussed, understood and as always, individualized with each patient."* |
|  | A position to be adapted according to the person | *"And the role of the patient is when we treat someone to tell us to express how they feel. How they are experiencing their treatment. Their life goals. Their quality of life goals as well. And so, it's really to be, to communicate and to express what he expects from the treatment and how he lives it and to guide us a little bit in the adaptation of all that"* |
| **Difficulties encountered in the care pathways:** | | |
| Organisational problems | Difficulties in coordination / transmission of information | *"And the orderlies, overall, the staff who do the care in continuous hospitalization or not are probably not very knowledgeable about the pathway. "* |
|  | Difficulties in communicating with the GP | *“The transmission of information with the general practitioner and that I think is the big difficulty."* |
|  | Difficulties in communication / coordination with out-of-hospital caregivers | *"For example, the private nurses receive prescriptions, but they have no visibility into the actual patient records, unless we give the records to the patients. And then it also requires extra time for her, time that is not accounted for. And the same goes for the attending physician. Of course, he receives all our letters, but on the other hand, he receives all the letters from all his patients... Does he have enough time to look into this? I don't think so because it's not a time that is counted, not reimbursed, not anything. I think the difficulties that can be there are there."* |
| **Suggestions for improving the care pathway** | | |
| Propose a referral professional | Suggestion to work in pairs between the referrer and another physician | *"I think one important thing is maybe companioning a senior who does the treatment changes, who is a referring physician, etc. And a junior, but one that would be fixed and that they would really see regularly."* |
| Creation of a pathway tracking tool | Well done and easy to use | *"Anything that tends to improve one's care pathway is a good idea. After that, the question is what tool? is it a materialized tool? dematerialized? It has to be an easy-to-use tool, for example, I see that health service providers have a transmission binder. Any help is good to take, but it must be well done and easy to use on both sides."* |
|  | Digital tools | *"So yes, digital tools, applications, things that are a little bit fun, not big pamphlets, where you give a sheet of information on both sides written in a very small format, with a whole bunch of information, some very important, very relevant, others much more blah. So here are things that are perhaps a little more fun.”* |
